# P12 ‘Pandemic preparedness’: training doctors for the next antimicrobial crisis

**DOI:** 10.1093/jacamr/dlaf230.019

**Published:** 2025-12-04

**Authors:** Miguel Vella, Li Ling Choo, Iram Lodhi

**Affiliations:** Frimley Health NHS Trust, Slough, UK; Frimley Health NHS Trust, Slough, UK; Frimley Health NHS Trust, Slough, UK

## Abstract

**Background:**

The COVID-19 pandemic revealed critical gaps in global healthcare readiness, including preparedness for antimicrobial resistance, a silent pandemic projected to cause 1.91 million annual deaths by 2050. Traditional medical education often fails to address the complex interplay between outbreak management and antimicrobial stewardship, leaving clinicians unprepared in dealing with emerging pathogens and treatment resistance.

**Objectives:**

To develop and evaluate an interactive simulation that trains medical students in outbreak response with particular focus on antimicrobial decision-making, identifying resistance patterns and stewardship principles during public health crises.

**Methods:**

The ‘Pandemic Preparedness’ curriculum was co-designed with infectious disease specialists, antimicrobial stewardship pharmacists and experts in undergraduate medical education. Five outbreak scenarios were created featuring high-consequence pathogens, most of which with significant resistance profiles. The five scenarios chosen were MDR TB, artemisinin-resistant malaria, a community outbreak of MRSA, *Clostridium difficile* in a hospital setting and an emerging strain of HIV with integrase inhibitor resistance. Each scenario was grounded in realistic constraints, including incomplete data, evolving case counts and regional socioeconomic variables. Students received case folders containing official communications (e.g. government briefings), annotated maps with demographic hotspots, case reports, microbiology reports and simulated news feeds to reflect public sentiment and misinformation. Some cases included dynamic antimicrobial resistance profiles that evolved based on treatment choices as well as simulated antimicrobial stock shortages requiring rational decision-making. Two scenarios incorporated simulated patients (mannikins with dynamic vitals) requiring bedside assessment, infection control measures and decision-making under time pressure. Students were also tasked with engaging in contact tracing and were allocated a constrained budget (e.g. £100 000) to choose between different interventions with real-time feedback on outbreak progression based on their choices.

**Results:**

‘Pandemic Preparedness’ has now been run three times among fifth and sixth year medical students from four universities. Overall, 88% of students (*n*=25) felt the teaching was relevant to their training. One hundred percent of participants expressed an increase in understanding about pandemic management. Participants were also asked to rank their confidence in various areas of study on a Likert Scale from 0–10 with 0 representing no confidence and 10 representing extreme confidence. Confidence in detecting and managing infectious diseases and outbreaks improved from 3.96 before the intervention to 7.80 after the intervention (97% improvement with a paired *t*-test *P* <0.0001) and confidence in selecting the appropriate antimicrobial therapy during outbreaks increased from 2.00 to 6.27 (214% improvement with a paired *t*-test *P* <0.0001). Finally, 100% of students said that they would recommend the programme to others.

**Conclusions:**

This simulation successfully integrates antimicrobial stewardship principles with outbreak response training, addressing a critical gap in medical education, with high student engagement and scalable adaptability for junior doctors.

